# PLAN: PREPARING AND LIVING FOR AGING NOW; A DESCRIPTIVE STUDY INVESTIGATING READINESS TO PLAN FOR AGING AND FRAILTY

**DOI:** 10.1093/geroni/igac059.095

**Published:** 2022-12-20

**Authors:** Erica Frechman, Cathy Maxwell, Mary Dietrich, Harleah Buck, Bethany Rhoten

**Affiliations:** Atrium Health Wake Forest, Concord, North Carolina, United States; Vanderbilt University, Hermitage, Tennessee, United States; Vanderbilt University, Nashville, Tennessee, United States; Csomay Center for Gerontological Excellence, Iowa City, Iowa, United States; Vanderbilt University School of Nursing, Nashville, Tennessee, United States

## Abstract

Population aging and the universality of the aging process offers individuals and society the opportunity to consider strategies to optimize older adults’ well-being and proactively prepare across their aging trajectory. Understanding aging through a holistic lens is essential to promote healthy aging in light of the risk for increasing chronic conditions, frailty, and disability that affect older adults. Planning for aging and frailty encompasses five domains that entail change as individuals age (communication/socialization, environmental, financial, physical care, cognitive status), and provides foci for a proactive approach to planning across life course. The aims of this research were to examine the stages of change through the Transtheoretical Model within the domains of aging, experiences (personal & experiences with others), and associations between contextual factors and stages of change for readiness to plan for aging and frailty. Using a cross-sectional design, 252 community dwelling-adults (aged 50-80) completed a survey on planning for aging and frailty. Results revealed a distribution of stages of change in readiness across domains of planning for aging and frailty with highest levels of planning in the financial domain (68.7%), and lowest levels in cognitive domain (28.2%). Participants reported increased experiences with others compared to personal experiences. Factors most indicative of planning include: older age, marital status, living situation, social support, and vulnerability. Planning for aging and frailty is an innovative concept that takes a comprehensive approach to the aging process. Future study is needed to promote planning across one’s life course and develop interventions focused on enhancing well-being.

